# Impact of age‐dependent red blood cell parameters on α‐globin gene genotyping in children

**DOI:** 10.1002/jha2.627

**Published:** 2022-12-13

**Authors:** Peter H. Nissen, Helene Narvestad‐Bøttger, Helle Pilgaard Kristensen, Anne Winther‐Larsen

**Affiliations:** ^1^ Department of Clinical Biochemistry Aarhus University Hospital Aarhus Denmark; ^2^ Department of Clinical Medicine Aarhus University Aarhus Denmark

**Keywords:** alpha‐thalassemia, genetic evaluation, hemoglobin A2, mean corpuscular hemoglobin, mean corpuscular volume, screening

## Abstract

When screening for α‐thalassemia in children, adult cut‐offs for mean corpuscular volume (MCV) and mean corpuscular hemoglobin (MCH) are generally applied to guide genetic evaluation. However, the normal ranges for MCV and MCH are lower in children than in adults, so we hypothesized that using age‐matched cut‐offs could lead to a more rational diagnostic strategy. The aim of this study was to evaluate if age‐matched cut‐offs could be applied advantageously. Data on children referred to a hemoglobin fractionation at the Department of Clinical Biochemistry, Aarhus University Hospital between 2016–2021 were identified in the laboratory information system. α‐globin gene (*HBA1/HBA2*) genotyping was performed using multiplex gap‐polymerase chain reaction. A total of 387 children were identified. *HBA1/HBA2*‐genotyping was performed in 207 children (53%), and α‐thalassemia was diagnosed in 47 children (23%) with −α^3.7^/αα being the predominant genotype (13%). We found that 23 children had MCV and MCH levels in the normal age‐matched range, and two of these children (9%) were α^+^ thalassemia carriers with three functional α‐globin genes. Using age‐specific cut‐off levels resulted in a reduction of 23 (11%) genotypes performed. In conclusion, applying age‐matched cut‐offs for MCV and MCH when screening children for α‐thalassemia lead to 11% fewer genotypes performed while 9% carriers of α^+^ thalassemia (of the medically innocuous genotype −α^3.7^/αα) would have been overlooked.

## 1 INTRODUCTION

α‐thalassemia is the most common monogenic genetic disorder worldwide [1]. The disorder results in a decreased or absent production of the α‐chain of hemoglobin, and the severity of the disorder is correlated to the number of affected α‐globin genes (*HBA1/HBA2*) [1]. Hence, α‐thalassemia is classified in two major subgroups as α^+^ thalassemia and α^0^ thalassemia. α^+^ thalassemia has one nonfunctional or deleted gene on one or two alleles and are predominantly the result of deletional variants removing one of two α‐globin gene copies on one allele (the ‐α^3.7^ and ‐α^4.2^ variants). α^0^ thalassemia is caused by deletional variants of both α‐globin genes on the same allele (e.g., the –^SEA^ and –^MED^ deletions) [1, 2].

As α‐thalassemia is protective against malaria [3], a high frequency of the disorder is found in malaria endemic areas including Africa, India, Southeast Asia, the Mediterranean, and the Middle East [4, 5, 6, 7]. Yet, owed to migration during the last several decades, the prevalence has increased in the rest of the world [8].

Based on the high prevalence of α‐thalassemia, it has been recommended to implement screening programs to identify carriers, and, in many countries, screening strategies at the premarital or early pregnancy level has been introduced [4, 9]. In these screening programs, mean corpuscular volume (MCV) and mean corpuscular hemoglobin (MCH) values are used to guide genetic evaluation, as α‐thalassemia carriers often present with microcytosis and are hypochromic; even though, α^+^ thalassemia heterozygotes may be hematologically silent [10]. Based on the normal ranges for MCV and MCH in adults, cut‐off levels of MCV < 78–80 fl and/or MCH < 27 pg/ < 1.68 fmol are widely used internationally when determining which patients to genotype [10, 11, 12, 13].

Despite screening programs, children are still born with α‐thalassemia. Hypocromia and microcytosis are typical biochemical findings in both iron deficiency anemia and α‐thalassemia, and therefore, α‐thalassemia genotyping in children with relevant biochemical findings and/or clinical symptoms is essential from a differential diagnostic point of view [14]. However, less information is available on the hematological parameters in children with α‐thalassemia [13, 15, 16] compared with adults. As a consequence, there seems to be a tendency to use the same cut‐offs for MCV and MCH for children as in adults when deciding which patients to genotype for α‐thalassemia [7, 17, 18], even though, it is well known that the normal ranges for these hematological parameters are lower in children. Hence, several children with MCV/MCH levels in the normal range for their age are selected for further genetic evaluation which probably entails the performance of numerous needless genetic tests for α‐thalassemia. Consequently, it is likely that age‐matched cut‐offs for MCV and MCH in children could lead to a more rational diagnostic strategy with a reduction in unnecessary α‐genotypes being performed. This would especially be of clinical relevance in areas with low prevalence of α‐thalassemia.

The aim of this study was to evaluate the hematological parameters according to α‐globin genotype in a cohort of children investigated for α‐thalassemia in a Danish laboratory. Moreover, we sought to evaluate if age‐matched cut‐offs based on reference intervals could be applied advantageously.

## 2 MATERIALS AND METHODS

### 2.1 Subjects and data collection

All children (<18 years) referred to a hemoglobin fractionation at the Department of Clinical Biochemistry, Aarhus University Hospital between October 2016 and September 2021 were identified in the clinical laboratory information system (LABKA II). All children with available results from the hemoglobin fractionation and *HBA* genotyping performed at the Department of Clinical Biochemistry were included. In each patient, sex, age, and laboratory results on red cell parameters, hemoglobin fractionation, and molecular analyses were retrieved from the laboratory system. Red cell parameters were only extracted if they were performed up to 1 week before the hemoglobin fractionation. If a hemoglobin fractionation had been performed several times in a child, only results from the earliest analysis were extracted.

In the routine screening program at our department, an *HBA* genotyping was performed if the child had a relevant ethnicity, MCV level <80 fl and/or MCH level <1.68 fmol, and there were no signs of iron deficiency assessed by ferritin level. If a ferritin value was unavailable and the two other criteria were met, an *HBA* genotyping was performed. In children with an HbA2 > 3.5%, a β‐globin gene (*HBB*) genotyping was performed. *HBA* and *HBB* genotyping were conducted independently of each other; thus, children with HbA2 > 3.5% and normal MCV/MCH according to adult cut‐off levels were only genotyped for *HBB* variants, while children with low MCV/MCH and normal HbA2 were only genotyped for *HBA* variants. Children with HbA2 > 3.5% and MCV/MCH below cut‐off level were genotyped for both *HBA* and *HBB* variants.

The study was approved by the local institutional board. According to the Danish law on ethics, requirement for informed consent was waived. The study was conducted in accordance with the Declaration of Helsinki.

### 2.2 Red blood cell parameters and hemoglobin fractionation

Red blood cell (RBC) parameters were evaluated by Sysmex XN9000 (Sysmex Corporation, Kobe, Japan) automated hematology systems. Briefly, the RBCs were analyzed using hydrodynamic focusing and impedance. Hemoglobin was analyzed using a sodium lauryl sulfate buffer and hemoglobin measured using spectrophotometry at 555 nm. We applied pediatric reference ranges for MCV and MCH from a recent study by Mrosewski et al. [19] (see Table S1), since they were performed on the same hematology analyzer as used in this study. In the oldest age groups, the partitioned reference intervals were combined to increase the group sizes.

The hemoglobin fractionation was performed by high‐performance liquid chromatography (HPLC) on a Tosoh G8 HPLC analyzer (Tosoh Corporation, Tokyo, Japan), utilizing cation‐exchange, using the β‐thalassemia mode according to the manufacturer's instructions.

### 2.3 Genotyping of HBA and HBB

Genomic DNA was isolated from EDTA stabilized whole blood using the Maxwell 16 Blood DNA Purification Kit (Promega, Nacka, Sweden) according to the manufacturer's instructions. After DNA isolation, the DNA was treated with RNase (Promega).

Genotyping of the α‐globin genes *HBA1* and *HBA2* was performed by multiplex gap‐PCR, essentially as described by Tan and co‐workers [20]. Briefly, primers representing seven different deletions in the α‐globin gene cluster (‐α^3.7^, ‐α^4.2^, –^SEA^, –^MED^, ‐(‐α)^20.5^, –^FIL^ and –^THAI^) were multiplexed, and PCR was performed in one PCR reaction on BioRad C1000 or S1000 thermocyclers (BioRad, Copenhagen, Denmark) using HotStar Taq (Qiagen, Copenhagen, Denmark). PCR conditions are available on request. PCR products were electrophoresed in 1.2% agarose gels, and genotypes were scored by two individuals.

*HBB* genotypes were established based on direct sanger sequencing of PCR products, as previously described [9], with minor modifications: PCR was performed using AmpliTaq Gold (Thermofisher, Roskilde, Denmark) in BioRad C1000 or S1000 thermocyclers (BioRad). The PCR products were purified using ExoSAP‐IT (Thermofisher), and sequenced using BigDye terminator version 1.1 (Thermofisher). Sequences were ethanol precipitated and separated on an Applied Biosystems 3500 or 3500XL Genetic Analyzer (Thermofisher). Sequence traces were aligned to the *HBB* reference sequence NM_000518 using SeqScape version 2.7 (Thermofisher).

Based on the number of functional α‐genes, patients were divided in four groups: 4 α: αα/αα; 3 α: ‐α^3.7^ /αα; 2 α: ‐α^3.7^ /‐α^3.7^, –^SEA^/αα, –^MED^/αα, ‐(α)^20.5^/αα; 1 α: –^SEA^ /‐α^3.7^, ‐(α)^20.5^/‐α^3.7^.

### 2.4 Statistical analysis

Data were tested for normality by visual inspection of histograms and Q‐Q plots and by use of Shapiro–Wilk test and were not found normally distributed. Thus, continuous parameters were presented as medians with interquartile ranges in brackets. The Kruskal–Wallis H test was used to compare groups. Statistical analyses were performed using SPSS statistics version 25.0 for Windows (IBM SPSS Statistics, Chicago, IL, USA). Graph art was performed using Graph Pad Prism version 9.3.1 (GraphPad Software Inc., California, USA).

## 3 RESULTS

**FIGURE 1:**
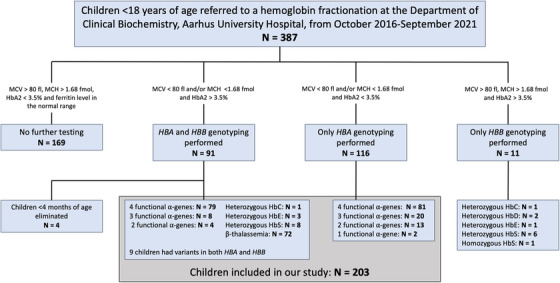
Flow chart of the screening and inclusion of children

A total of 387 children were identified (205 girls and 182 boys) with a median age of 9 years (range 3 days to 17 years). The median hemoglobin level in all boys was 7.3 mmol/L (6.2‐8.2) and 7.1 mmol/L (6.2‐7.8) in girls. In all children, the median MCV was 75 fl (63‐83), the median MCH was 1.49 fmol (1.19‐1.72), the median MCH concentration (MCHC) was 19.9 mmol/L (19.0‐20.6), and the median HbA2 was 2.7% (2.2‐3.2). A flow chart showing the selection of children, and the screening criteria are depicted in Figure 1.

According to the MCV and/or MCH levels, 207 children (53%) were referred to *HBA* genotyping, and an α‐thalassemia was diagnosed in 47 children (23%). As seen in Table 1, seven different α‐genotypes were identified with ‐α^3.7^/αα being the predominating genotype constituting 13% of all α‐thalassemia carriers.

**TABLE 1:** Genotypes in children evaluated with molecular analysis of the α‐globin genes (*N* = 203)

α‐globin genotype	*N* (%)
αα/αα	157 (77)
‐α^3.7^ /αα	27 (13)
‐α^3.7^ /‐α^3.7^	8 (4)
–^SEA^/αα	4 (2.0)
–^MED^/αα	4 (2.0)
‐(α)^20.5^/‐α^3.7^	1 (0.5)
‐(α)^20.5^/αα	1 (0.5)
–^SEA^ /‐α^3.7^	1 (0.5)

### 3.1 RBC indices based on HBA genotype

**FIGURE 2:**
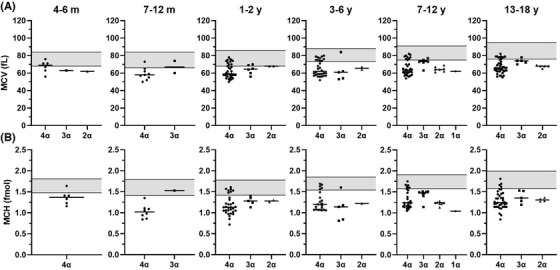
Mean corpuscular volume (MCV) and MCH levels according to the number of functional α‐genes and the age of the children genotyped for α‐thalassemia (*N* = 203). Age is indicated at the top of the figure. Median values are shown as horizontal lines. Reference intervals for each age group are shown as grey boxes. (A) MCV; reference intervals: 0–3d: 92.5–111.4 fl; 3 m: 71.6–87.0 fl; 4–6 m: 67.9–84.3 fl; 7–12 m: 66.3–83.8 fl; 1–2y: 67.9–85.8 fl; 3–6y: 72.5–88.2 fl; 7–12y: 74.9–90.5 fl; 13–18y: 75.6–95.2 fl. (B) MCH; reference intervals: 0–3d: 2.05–2.43 fmol; 4–6 m: 1.48–1.81 fmol; 7–12 m: 1.41–1.79 fmol; 1–2y: 1.42–1.78 fmol; 3–6y: 1.54–1.85 fmol; 7–12y: 1.58–1.91 fmol; 13–18y: 1.58–1.99 fmol. d, days; m, months; MCH, mean corpuscular hemoglobin; y, years

Values for MCV, MCH, and HbA2 were missing in 3, 35, and 2 children, respectively. As only four children were below 4 months of age, and only one of them was an α‐thalassemia carrier, these children were excluded from further analyses. The level of hemoglobin, MCV, and MCH according to the number of functional α‐globin genes and the age of the children are shown in Table 2 and Figure 2 for the 203 children genotyped for α‐thalassemia. In children aged 7–18 years, a decrease in median MCV and MCH level was observed when comparing α‐thalassemia carriers with three functional α‐globin genes with carriers with two functional α‐globin genes (Table 2 and Figure 2). This tendency was not observed in children aged 1–6 years. Overall, a large overlap was seen between the non‐α‐thalassemia children (four functional α‐globin genes) and the carriers of α‐thalassemia.

**TABLE 2:** The level of hemoglobin, MCV, and MCH according to the number of functional α‐genes subdivided by age for the 203 children genotyped for α‐thalassemia

		Hemoglobin (mmol/L)			MCV (fl)			MCH (fmol)		
Age	Func‐tional α‐genes	Normal range^†^	*N*	Median (IQR)	Normal range^†^	*N*	Median (IQR)	Normal range^†^	*N*	Median (IQR)
4–6 m	4 α	6.3–8.1	6	7.1 (6.4–7.8)	68‐84	6	69 (62–71)	1.48–1.81	6	1.37 (1.22–1.45)
	3 α		1	6.1		1	63			
	2 α		1	6.9		1	62			
	1 α									
7–12 m	4 α	6.1–8.4	9	5.2 (4.6–5.7)	66–84	9	58 (54–63)	1.41‐1.79	8	1.02 (0.89–1.10)
	3 α		2	5.8 (5.5–6.1)		2	67 (60–74)		1	1.53
	2 α									
	1 α									
1–2y	4 α	6.3–8.4	36	6.1 (5.0–7.2)	68–86	36	59 (56–70)	1.42‐1.78	32	1.13 (1.03–1.34)
	3 α		6	6.9 (6.5–7.2)		6	65 (60–69)		5	1.28 (1.20–1.37)
	2 α		2	6.7 (6.5–6.8)		2	68 (68–68)		2	1.28 (1.26–1.30)
	1 α									
3–6y	4 α	6.8–8.7	30	6.8 (6.2–7.3)	73–88	30	62 (58–75)	1.54–1.85	21	1.20 (1.07–1.51)
	3 α		5	5.6 (4.0–6.4)		5	61 (54–69)		5	1.14 (0.83–1.32)
	2 α		2	7.2 (7.1–7.3)		2	66 (64–67)		1	1.22
	1 α									
7–12y	4 α	7.3–9.2	32	6.9 (6.3–7.7)	75–91	32	64 (60–76)	1.58–1.91	28	1.24 (1.14–1.56)
	3 α		8	7.1 (6.8–7.6)		8	74 (72–75)		8	1.48 (1.43–1.51)
	2 α		7	7.2 (6.8–7.5)		7	64 (62–67)		7	1.24 (1.22–1.25)
	1 α		2	5.9 (5.5–6.2)		1	62		1	1.04
13–18y	4 α	7.0–10.7	42	7.0 (6.5–7.5)	76‐95	42	66 (62–75)	1.58–1.99	37	1.24 (1.14–1.44)
	3 α		5	6.8 (5.5–7.6)		5	74 (71–75)		5	1.35 (1.25–1.52)
	2 α		5	7.9 (7.2–8.5)		5	68 (67–68)		4	1.31 (1.27–1.35)
	1 α									

Abbreviations: d, days; IQR, interquartile range; m, months; MCH, mean corpuscular hemoglobin; MCV, mean corpuscular volume; n, number; y, years.

^†^ Reference intervals modified from Mrosewski et al. [19], see Table S1.

The 203 children were divided according to their MCV and MCH level being below or above the lower reference limit for MCV and MCH in their age group and the number of functional α‐globin genes. As shown in Table 3, 53 children had MCV levels in the normal age‐matched range of whom nine children (9/53, 17%) had α‐thalassemia. Likewise, 25 children had an MCH level in the reference interval, and three of these children (3/25, 12%) were α‐thalassemia carriers.

**TABLE 3:** Children genotyped for α‐thalassemia, divided according to MCV and MCH levels being above or below the lower reference range for MCV and MCH in their age group and the number of functional α‐genes

			Functional α‐genes		
	Total	4 α	3 α	2 α	1 α
MCV^†^					
<lower reference limit	149	113	20	15	1
>lower reference limit	53	44	7	2	0
Total	202	157	27	17	1
MCH^†^					
<lower reference limit	146	110	22	13	1
>lower reference limit	25	22	2	1	0
Total	171	132	24	14	1
MCV and MCH^†^					
<lower reference limit	125	96	17	11	1
>lower reference limit	23	21	2	0	0
Total	148	117	19	11	1

Abbreviations: MCH, mean corpuscular hemoglobin; MCV, mean corpuscular volume.

^†^ Reference intervals modified from Mrosewski et al. [19], see Table S1.

Altogether, 23 children had normal age‐matched levels of both MCV and MCH, whereof two (2/23, 9%) were α^+^ thalassemia carriers with three functional α‐genes.

Thus, using age‐matched lower reference ranges for MCV and MCH when screening children would lead to a reduction of 23 (23/203, 11%) fewer genotypes performed; however, the diagnosis of two α^+^ thalassemia of the genotype –α^3.7^/αα would have been overlooked.

### 3.2 HbA2 level and β‐thalassemia

Based on an elevated HbA2 level above 3.5%, *HBB* sequencing was performed in 102 children (26%) (Figure 1). Hemoglobin variants were identified in 96 of these children (94%), representing carriers of β‐thalassemia and both hetero‐ and homozygous states of HbS, HbE and carriers of HbD and HbC. Coexisting α‐ and β‐thalassemia was found in six children, and three children concurrently carried α‐thalassemia and a hemoglobin variant (one HbE, one HbS, and one HbC).

The HbA2 level was evaluated in the α‐genotyped children excluding the children diagnosed with either β‐thalassemia or a hemoglobin variant as well as children below 6 months, as the HbA2 can be undetectable or very low at birth, and does not become stable until 6 months of age [21] (*N* = 115). As shown in Table S2, the HbA2 level was very similar in the non‐α‐thalassemia group (*N* = 80) and in the α‐thalassemia children with two and three functional α‐genes (*N* = 33) while the HbA2 level in the two children with only one functional α‐gene was very low. Yet, no overall statistically significant difference was found between the different groups (Kruskal–Wallis H test = 5.75, *p* = 0.120). The HbA2 level was ≤3.0% in all the α‐thalassemia children with no concomitant β‐thalassemia or hemoglobin variant.

## 4 DISCUSSION

In children with relevant ethnicity, α‐thalassemia is an important differential diagnosis to iron deficiency for hypochromic, microcytic anaemia. Over a 5‐year period, 387 children were referred by clinicians to a hemoglobin fractionation at our laboratory, supporting the relevance of examining children under suspicion for a haematological disorder. However, often the use of adult cut‐off levels for MCV and MCH are applied when evaluating children prior to molecular diagnosis for α‐thalassemia, even though the normal ranges of MCV and MCH throughout most of childhood are lower in children than in adults. This could lead to numerous needless genotypes performed. Therefore, we evaluated if age‐matched cut‐offs for MCV and MCH in children could lead to a more rational diagnostic strategy with a reduction in unnecessary α‐genotypes without the risk of overlooking carriers, and observed that using age‐matched cut‐offs in the 203 children included in our study would have led to 23 fewer genotypes performed (11%). However, this came at the expense of two children with silent α^+^ thalassemia (three functional genes) (9%) being undiagnosed.

In adults, it is well known that carriers of α^+^ thalassemia can be hematologically silent and that a particular overlap in MCV and MCH are seen for α‐thalassemia carriers and non‐α‐thalassemia individuals. In a recent study from China, including 13,294 adult subjects evaluated for α‐thalassemia, 21% of α‐thalassemia carriers had MCV > 80 fl and 14% had MCH > 27 pg [22]. Thus, using these standard cut‐offs for MCV and MCH for guiding genotyping would have led to 14% of carriers being missed. Our study was performed on a selected population of children under suspicion for a hematological disorder in contrast to the population in the Chinese study; however, taking into account that an undiscovered diagnosis of a hematologically silent α^+^ thalassemia carrier with only one α‐gene missing is not medically problematic, it seems acceptable to have a diagnostic set‐up that misclassify 9% of these children.

We observed a large overlap in MCV and MCH between the carriers of α‐thalassemia and the non‐α‐thalassemia children. This is in line with a recent Italian study by Origa et al. [13] including 453 children where a significant overlap in MCV, MCH, and hemoglobin was seen between α^+^ thalassemia heterozygous children and normal subjects and α‐thalassemia children with only two functional α‐globin genes, respectively. Yet, in contrast to our study, Origa et al. found no overlap for MCV and MCH between children with two α‐globin gene defects and normal children. In our study, the ranges of MCV and MCH levels in the non‐α‐thalassemia children were very wide, probably reflecting that a portion of the non‐α‐thalassemia children had other hematological conditions affecting their hematological parameters, as all the children in our study were referred to a hemoglobin fractionation based on a clinical suspicion of disease. This may explain the observed discrepancy between the MCV and MCH levels found in the non‐α‐thalassemia children in the two studies. We found a decrease in median MCV and MCH when comparing children with three versus two functional α‐genes, but only for children aged 7–18 years. In the study by Origa et al. [13], this decrease was found for all ages (0.5–15 years). However, when partitioned by number of functional α‐genes in our study, we had limited data in each age group, which might explain why this tendency was not observed for children of all ages.

Interestingly, two children with HbH disease (one functional α‐gene) were identified in our cohort. People with HbH disease are often healthy as children but are at risk of developing hemolytic crises and chronic complications as they age, meaning the condition is very important to diagnose from a medical perspective. Both children with HbH presented with very low MCV/MCH values and were selected for *HBA* genotyping based on the age‐matched cut‐offs, indicating that this approach may identify clinically important cases like HbH disease.

Aside from the improved cost‐efficiency of α‐thalassemia genotyping in children when applying age‐matched cut‐offs, the risk of overlooking α‐thalassemia in newborns is also decreased. It is well‐established that the normal levels of MCV and MCH are higher in newborns compared to adults [13, 23], meaning some newborns could be misclassified if their MCV/MCH level were above the lower limit for adults, but below the lower limit for newborns.

When excluding children diagnosed with either β‐thalassemia or a β‐globin variant, the median HbA2 level did not vary between the α‐thalassemia children and the non‐α‐thalassemia children, except in the two children with HbH disease who had considerably lower HbA2 levels than the non‐α‐thalassemia children (1.2 [1.0–1.3] versus 2.4 [2.1–2.7]). This finding is in contrast to Origa et al. [13] who observed lower HbA2 levels in all α‐thalassemia carriers compared with non‐α‐thalassemia controls independent of the number of functional α‐genes. Data on adult α‐thalassemia carriers have shown HbA2 levels below or low in the normal range with a large overlap in levels between carriers and noncarriers [10, 22]. Furthermore, very low HbA2 levels have been found in adults with HbH disease [24], and it is possible that the same could be expected in children. However, the normal ranges of HbA2 in children are yet to be defined, making it even more difficult to interpret the levels observed.

We identified seven different α‐globin genotypes in the children. The –α^3.7^/αα and the –α^3.7^/–α^3.7^ dominated with 60% and 17% of the identified genotypes, respectively. This corresponds well with results from previous studies showing that the –α^3.7^ deletion is the most common [1, 9].

The present study has some limitations to consider. Even though we included all children from a 5‐year period, the number of children was limited, as we had to group them according to age and functional α‐genes, reducing the number of children in each group considerably. Furthermore, as our study is a real‐world setting including children referred to hemoglobin fractionation at our department, all the included children were under suspicion for a hematological disorder. Additionally, as a ferritin measurement was not available in all of the included children, it is possible that some of the children had iron deficiency that we were not able to diagnose. Therefore, the non‐α‐thalassemia group was not a control group of healthy children. Hence, we were unable to compare the level of the hematological parameters of the α‐thalassemia children with healthy controls in this study. Additionally, the laboratory methods were restricted to the methods used in the diagnostic set‐up for screening adults as part of the screening program, that is, multiplex gap‐PCR identifying seven *HBA* deletions. Thus, sequence variants of the *HBA* genes were not analyzed. Lastly, information on ethnicity was not recorded, hampering the ability to evaluate the frequency of the different deletions according to ethnicity.

In conclusion, applying age‐matched cut‐off levels for MCV and MCH in guiding α‐thalassemia genotyping in children led to 11% fewer genotypes performed with only very few α^+^ thalassemia children of the medically innocuous genotype –α^3.7^/αα being overlooked. If our data can be confirmed in a larger cohort, this strategy could lead to a more rational diagnostic strategy with a reduction in unnecessary α‐genotypes performed.

## AUTHOR CONTRIBUTIONS

All authors performed the research. PHN, HPK, and AWL designed the research study. HNB and AWL analyzed the data. AWL wrote the first draft of the paper. All authors have read and agreed to the published version of the manuscript.

## CONFLICT OF INTEREST

The authors state no conflict of interest.

## FUNDING INFORMATION

The authors received no specific funding for this work.

## Supporting information

Supporting Information

## Data Availability

The data that support the findings of this study are available on request from the corresponding author. The data are not publicly available due to privacy or ethical restrictions.
