# Supplementary material for: Impact of age‐dependent red blood cell parameters on α‐globin gene genotyping in children
Source: EJHaem. 2022 Dec 13;4(1):18–25. doi: 10.1002/jha2.627 (PMC9928656; doi:10.1002/jha2.627)
Supplement: Supplementary file 1 — Supporting Information [file JHA2-4-18-s001.docx]

Supplementary Table 1: Pediatric reference ranges for haemoglobin, MCV and MCH used in our study based on Mrosewski et al. (19)

| **Age** | **Hemoglobin, mmol/L** | **MCH, fmol** | **MCV, fL** |
| --- | --- | --- | --- |
| 4-6m | 6.3-8.1 | 1.48-1.81 | 67.9-84.3 |
| 7-12m | 6.1-8.4 | 1.41-1.79 | 66.3-83.8 |
| 1-2y | 6.3-8.4 | 1.42-1.78 | 67.9-85.8 |
| 3-6y | 6.8-8.7 | 1.54-1.85 | 72.5-88.2 |
| 7-12y | 7.3-9.2 | 1.58-1.91 | 74.9-90.5 |
| 13-18y^†^ | 7.0-10.7 | 1.58-1.99 | 75.6-95.2 |
| 13-18y, Female | 7.0-9.3 | 1.60-1.99 | 77.9-95.2 |
| 13y, Male | 7.6-9.8 | 1.58-1.92 | 75.6-91.3 |
| 14-15y, Male | 7.8-10.4 | 1.59-1.95 | 76.7-92.8 |
| 16-18y, Male | 8.3-10.7 | 1.66-1.99 | 79.1-94.1 |

Abbreviations: d, days; m, months; y, years.

^†^ In the study by Mrosewski et al., the reference interval for 13-18y was originally divided by sex, and for boys also divided in three age groups, see grey part of the table. However, we have modified the reference intervals by combining them in the present study, taking the lowest value as lower limit and the highest value as upper limit.

Supplementary Table 2: The HbA2 level according to number of functional α-genes in children older than 6 months genotyped for α-thalassemia, excluding the children diagnosed with either β-thalassemia or a hemoglobin variant (N=115).

| **Number of functional α-genes** | **N** | **HbA2 (%)** | | |
| --- | --- | --- | --- | --- |
|  |  | **Median (IQR)** | **Range** | **P-value** |
| **4 α** | 80 | 2.4 (2.0-2.7) | 1.0-3.4 | 0.120* |
| **3 α** | 19 | 2.5 (2.2-2.7) | 1.2-3.0 |  |
| **2 α** | 14 | 2.3 (2.1-2.5) | 1.9-3.0 |  |
| **1 α** | 2 | 1.2 (1.0-1.3) | 1.0-1.3 |  |

Abbreviations: IQR, interquartile range.

*P-value was calculated with the Kruskal-Wallis H test.
